# Supplementary material for: MADS1-regulated lemma and awn development benefits barley yield
Source: Nat Commun. 2024 Jan 5;15:301. doi: 10.1038/s41467-023-44457-8 (PMC10770128; doi:10.1038/s41467-023-44457-8)
Supplement: Supplementary file 1 — Supplementary Information [file 41467_2023_44457_MOESM1_ESM.pdf]

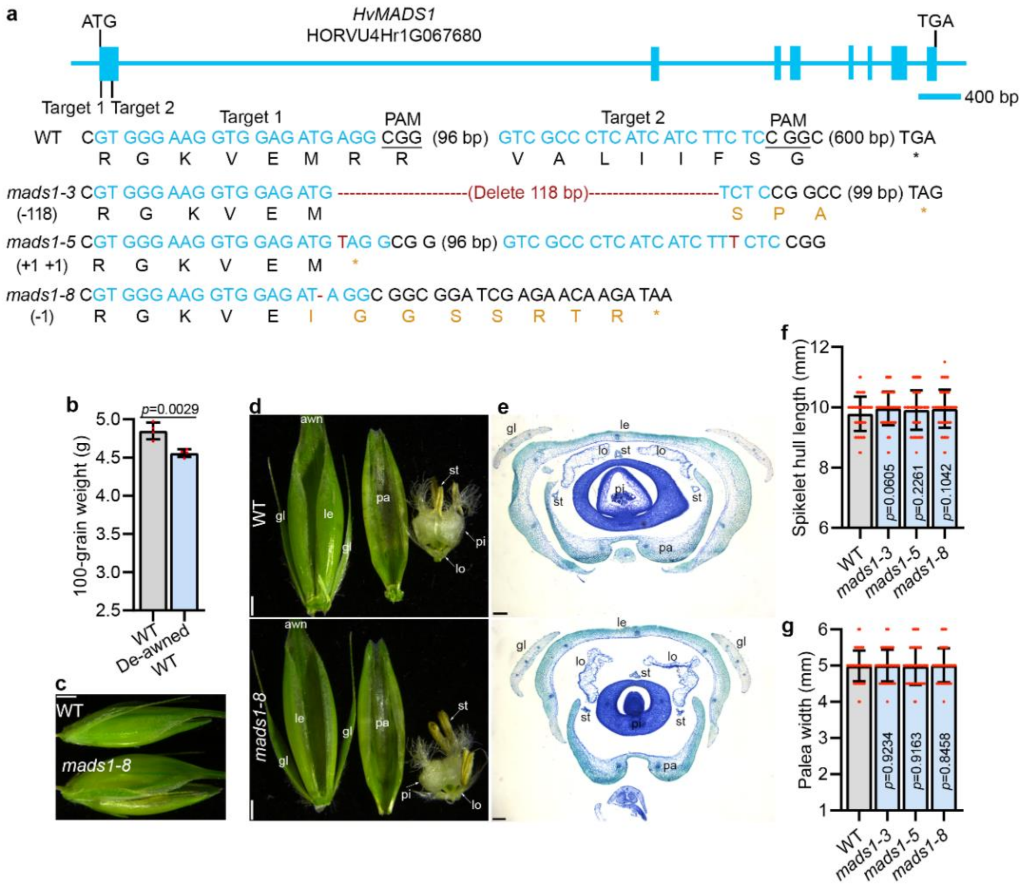


**Supplementary Fig. 1 Phenotypic analysis of WT and *mads1* spikelets.**

**a** Knockout mutation in the *HvMADS1* gene induced by CRISPR/Cas9. Gene schematic shows exons (blue boxes), introns (blue line between two neighboring exons) and untranslated regions (blue line before ATG and after TGA), start and stop codons (ATG and TGA, respectively), and positions of CRISPR/Cas9 targets 1 and 2 in exon 1 (light blue letters). *mads1-3*, *mads1-5* and *mads1-8* had a 118- base pair (bp) deletion, two 1-bp insertions, and a 1-bp deletion, respectively, as shown in light red dashed line. All alleles caused a premature stop codon (light orange asterisk). PAM, protospacer adjacent motif. Underlined letters indicate PAM sequences. **b** Effect of de-awn on grain weight in wild type (WT). **c** A image of WT and *mads1* spikelet, respectively. **d** A dissected WT and *mads1* spikelet showing separated lemma and palea, respectively. **e** Transverse sections of a WT (upper) and *mads1* (lower) spikelet, showing similar morphologies. **f, g** Statistic data of spikelet hull length and palea width of WT and *mads1*, respectively. Values are mean ± SD, *p* values obtained from two-tailed Student's t-test; Scale bars, 1 mm (**c**, **d**), 100 µm (**e**). gl, glume; le, lemma; lo, lodicule; pa, palea; pi, pistil; st, stamen. Source data are provided as a Source Data file.

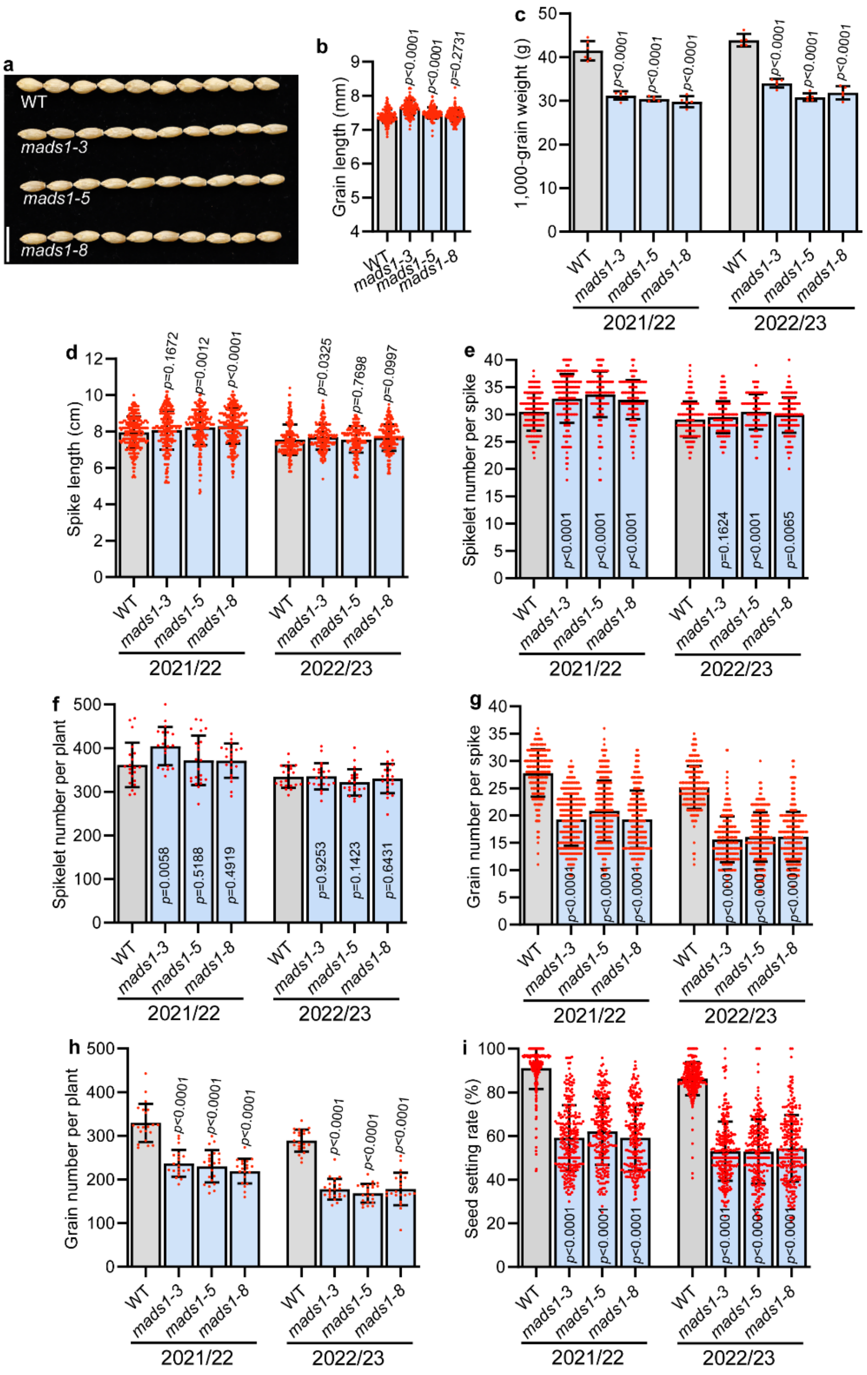


**Supplementary Fig. 2 Spikelet and grain related traits of WT and *mads1*.**

**a, b** Images (**a**) and statistical data (**b**) of WT and *mads1* grain length. **c–i** 1,000-grain weight (**c**), spike length (**d**), spikelet number per spike (**e**), spikelet number per plant (**f**), grain number per spike (**g**), grain number per plant (**h**), and seed setting rate (**i**) of WT and *mads1* grown in the paddy field in Shanghai for two consecutive years (2021/22 and 2022/23). Values are mean ± SD, *p* values obtained from two-tailed Student's t-test; red dots give actual results from individual replicates. Scale bars, 1 cm (**a**). Source data are provided as a Source Data file.

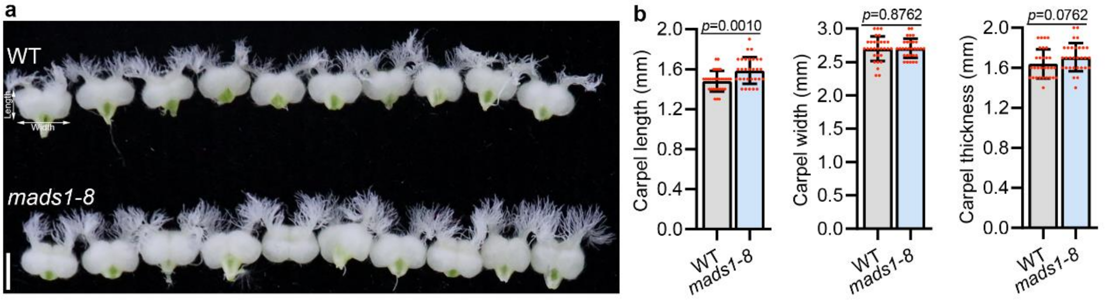


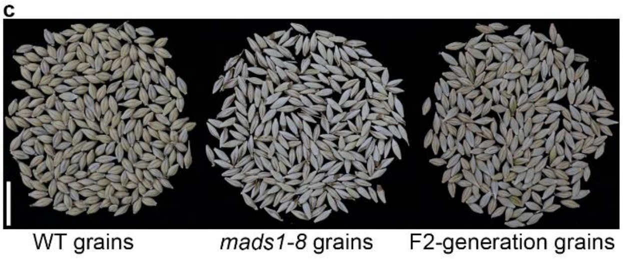


d

| *mads1-8* (♀) cross WT (♂) | # (%) of normal grain in F2 | # (%) of small grain in F2 |
|---|---|---|
| Repeat 1 | 235 (100 %) | 0 (0 %) |
| Repeat 2 | 106 (100 %) | 0 (0 %) |
| Repeat 3 | 253 (100 %) | 0 (0 %) |

**Supplementary Fig. 3. Effects of carpel and endosperm on grain size.**

**a** Images of WT and *mads1* carpels. **b** Statistic data of carpel length, carpel width and carpel thickness. **c** Grain images of the offsprings of F1 generated by crossing *mads1-8* (♀) with WT (♂). **d** Statistic data of normal and small grain in the offspring of F1 generated by crossing *mads1-8* (♀) with WT (♂). Values are mean ± SD, *p* values shown are from two-tailed Student's t-test (n = 30 individual carpels). Scale bars, 2 mm (**a**) and 2 cm (**c**). Source data are provided as a Source Data file.

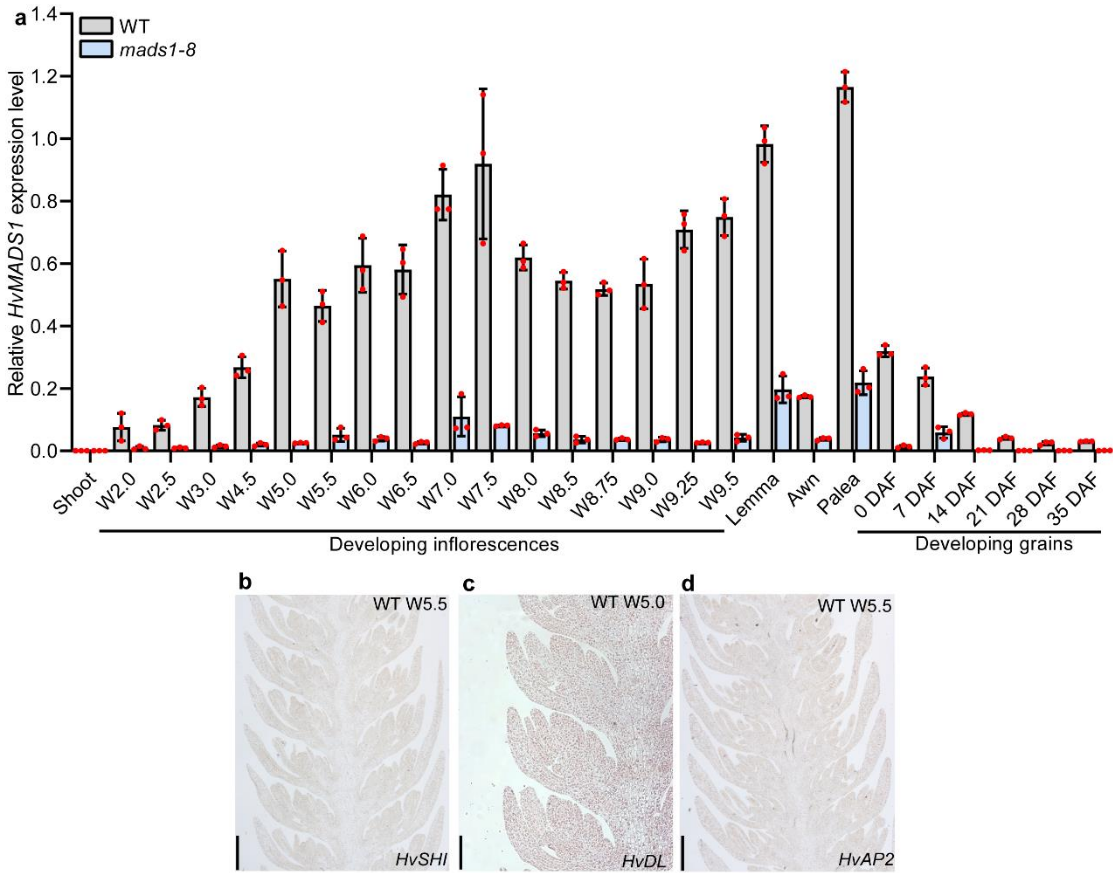


**Supplementary Fig. 4 Spatiotemporal expression pattern of *HvMADS1* in WT and *mads1* and *in situ* hybridization result of several genes in WT with sense probes.**

**a** Expression of *MADS1* in different tissues, particularly in developing inflorescences and developing grains, of WT and *mads1*. **b–d** *In situ* hybridization results detected with sense probes of *HvSHI* (**b**), *HvDL* (**c**) and *HvAP2* (**d**), in developing WT inflorescences. Values are mean ± SD (n = 3 biological replicates). Scale bars, 100 μm (**b**, **d**), 250 μm (**c**). DAF, days after fertilization. Source data are provided as a Source Data file.

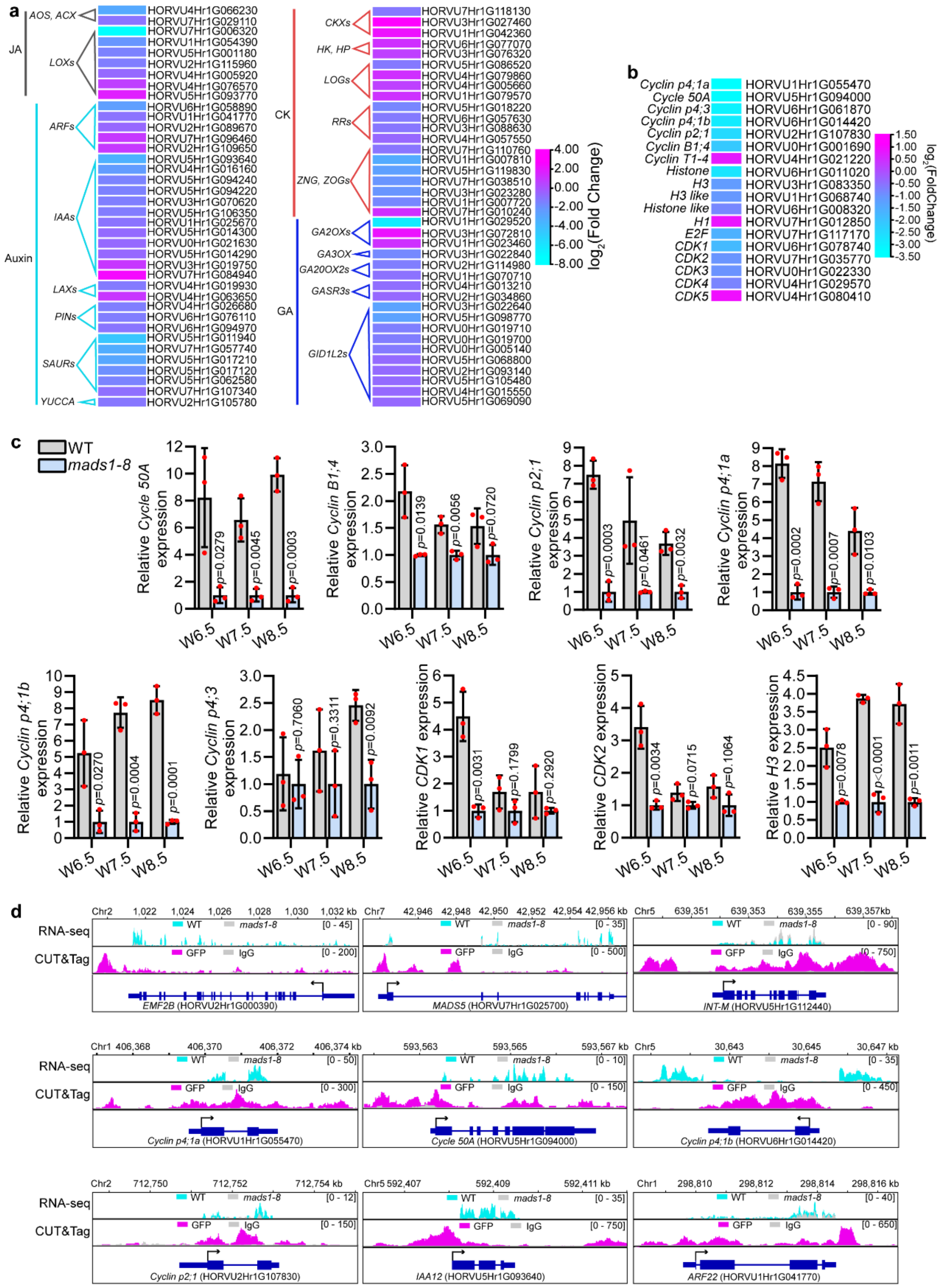

**Supplementary Fig. 5. DEGs related to hormones, cell cycle and floral organ development.**

**a**, **b** Heatmap of expression of hormone-related genes (**a**) and cell cycle-related genes (**b**) altered in the *mads1* lemmas and awns identified by RNA-seq. **c** Validation of the expression of selected cell cycle-related genes in WT and *mads1* lemmas and awns by RT-qPCR. **d** Some putative target genes

of HvMADS1 identified by RNA-seq and CUT&Tag. Genome browser views of RNA-seq and CUT&Tag profiles around genes involved in floral organ development (*EMF2B*, *MADS5*, *INT-M*), cell-cycle (*Cyclins, Cycle 50A*) and auxin (*IAA12, ARF22*). ACX, Acyl-coenzyme A oxidase; AOX, Allene oxide synthase; ARF, Auxin response factor; CK, cytokinin; CDK, cyclin-dependent protein kinase; CKX, cytokinin oxidase; EMF2B, EMBRYONIC FLOWER 2B; GA, gibberellic acid; GA2OX, GA2 oxidase; GA3OX, GA3 oxidase; GA20OX2, GA20 oxidase 2; GFP, green fluorescent protein antibody; GASR3, gibberellin-regulated family protein 3; GID1L2, alpha/beta-hydrolases superfamily protein; H3, histone H3; HK, histidine kinase; HP, histidine acid phosphatase; IAA, auxin/indole-3-acetic acid; IgG, immunoglobulin antibody; INT-M, INTERMEDIUM-M; JA, jasmonic acid; LAX, auxin transporter-like protein; LOG, lysine decarboxylase family protein; LOX, lipoxygenase; PIN, auxin efflux carrier family protein; RR, response regulator; SAUR, SAUR-like auxin-responsive protein family; YUCCA, flavin monooxygenase; ZOG, CK O-glucoside; ZNG, CK N-glucoside; Values are means ± SD, $p$ values obtained from two-tailed Student's t-test (n = 3 biological replicates). Source data are provided as a Source Data file.

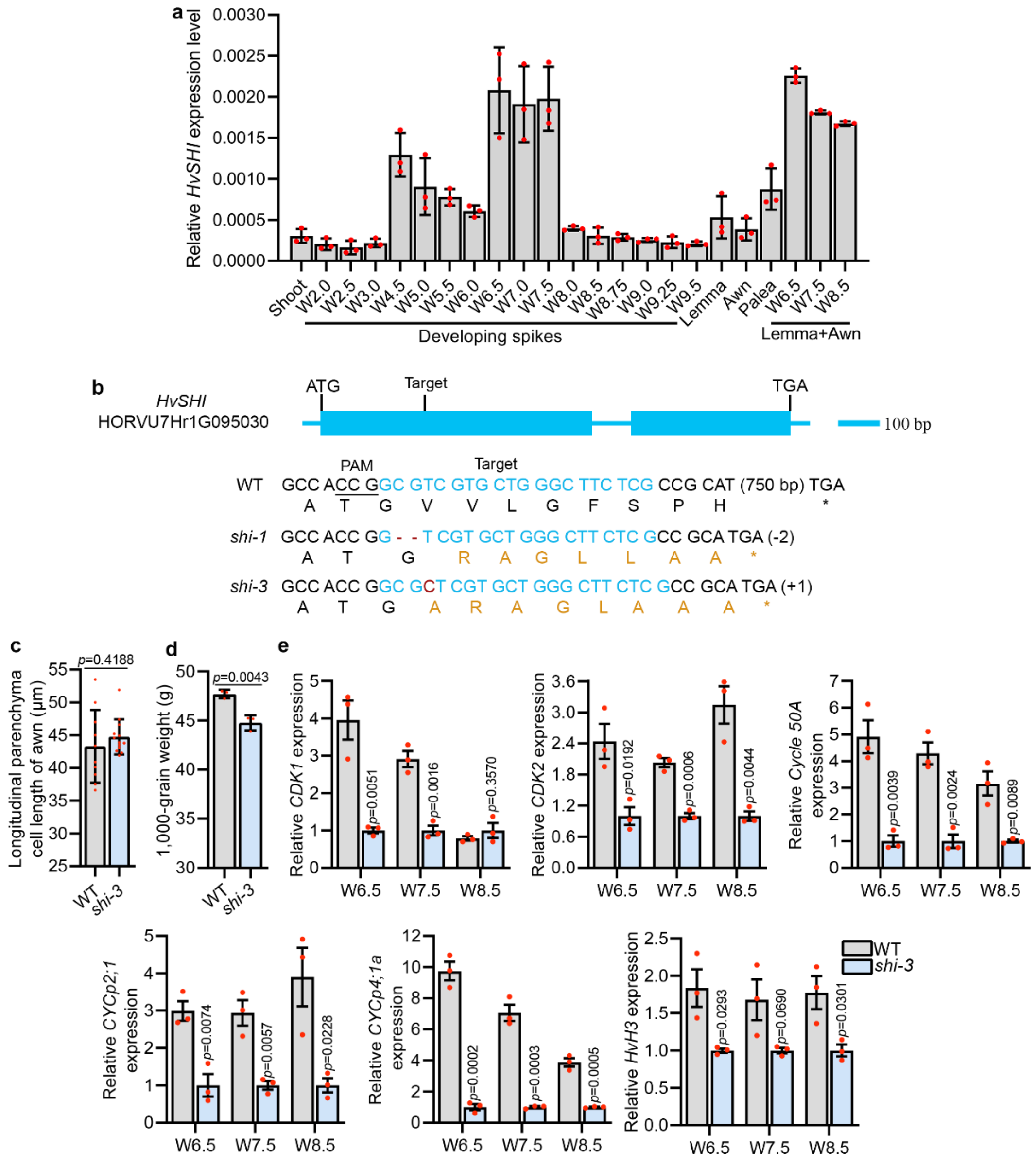


**Supplementary Fig. 6. Functional characterization of *HvSHI*.**

**a** Spatiotemporal expression patterns of *HvSHI* as revealed by RT-qPCR. **b** Creation of *shi* knockout mutant lines using CRISPR/Cas9. Two different mutant alleles were created, *shi-1* (2- bp deletion, light red dashed line) and *shi-3* (1- bp insertion, light red letter), each producing a premature stop codon (light orange asterisk). PAM, protospacer adjacent motif. **c** Statistic data of longitudinal parenchyma cell length in *shi* awns. **d** Statistic data of 1,000-grain weight of WT and *shi*. **e** Expression of six tested cell cycle genes in WT and *shi* lemmas and awns detected by RT-qPCR. Values are mean ± SD, *p* values obtained from two-tailed Student's t-test (n = 3 biological replicates for **a, d, e**). Source data are provided as a Source Data file.

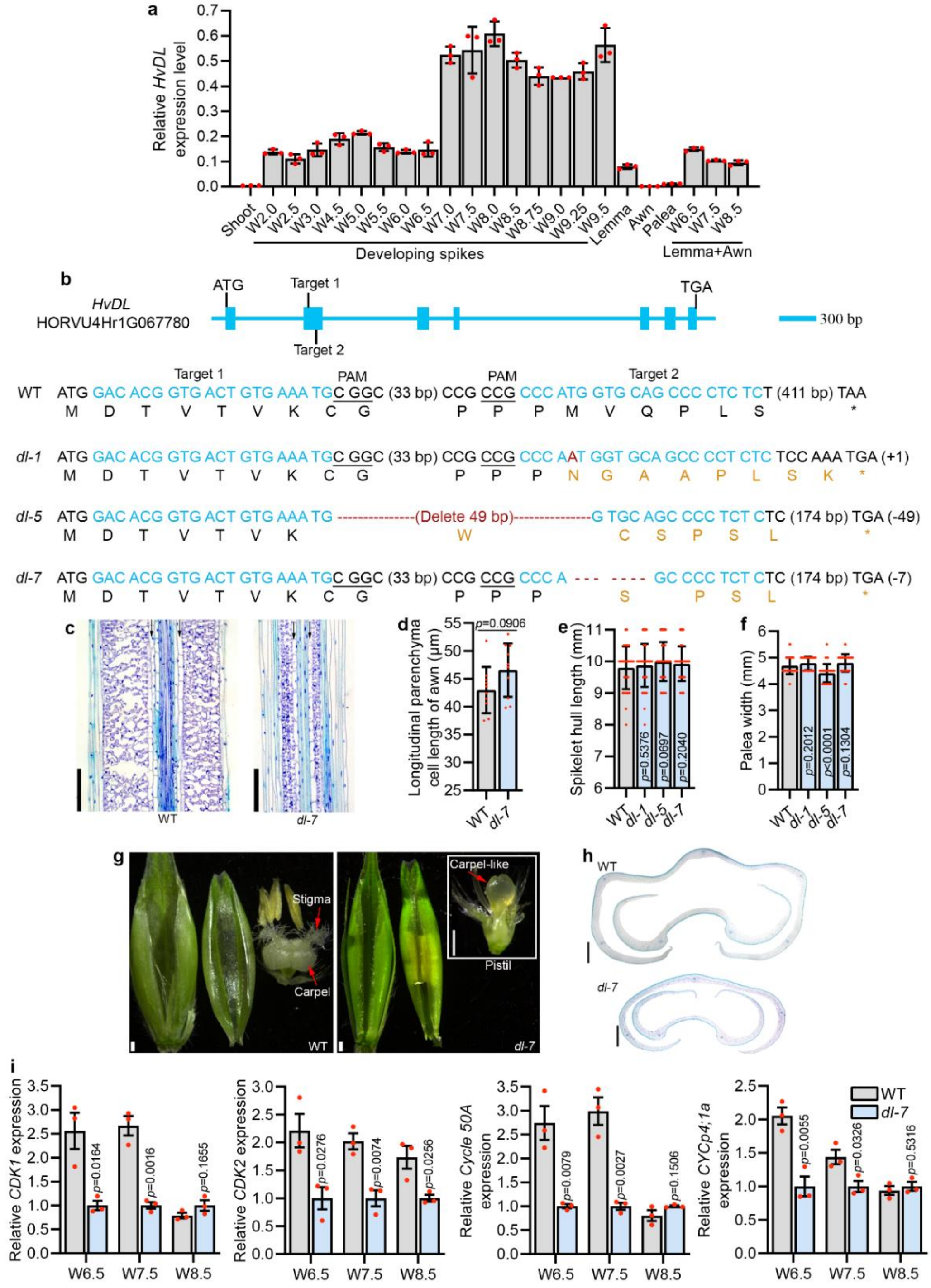

**Supplementary Fig. 7. Functional characterization of *HvDL*.**

**a** Spatiotemporal expression patterns of *HvDL* as revealed by RT-qPCR. **b** Creation of *dl* knockout mutant line created using CRISPR/Cas9. Three different mutant alleles were created, *dl-1* (1- bp insertion, light red letter), *dl-5* (49- bp deletion, light red dashed line), and *dl-7* (7- bp deletion, light

red dashed line), each producing a premature stop codon (light orange asterisk). PAM, protospacer adjacent motif. **c** Longitudinal sections from boxed regions of Fig. 5o, showing cell size of WT and *dl* awns. Black arrows indicate cells used to measure cell length and cell number. **d–f**, Statistic data of the length of longitudinal parenchyma cells in awns (**d**), spikelet hull length (**e**), and palea width (**f**) in WT and *dl*. **g** Pistil phenotypes of WT and *dl* mutant. **h** A cross-section of WT (upper) and *dl* (lower) spikelet hulls, respectively. **i** Expression of four tested cell cycle genes in WT and *dl* lemmas and awns determined by RT-qPCR (n = 3 biological replicates). Values are means ± SD, *p* values obtained from two-tailed Student's t-test. Scale bars, 100 μm (**c**), 500 μm (**g**), 250 μm (**h**). Source data are provided as a Source Data file.

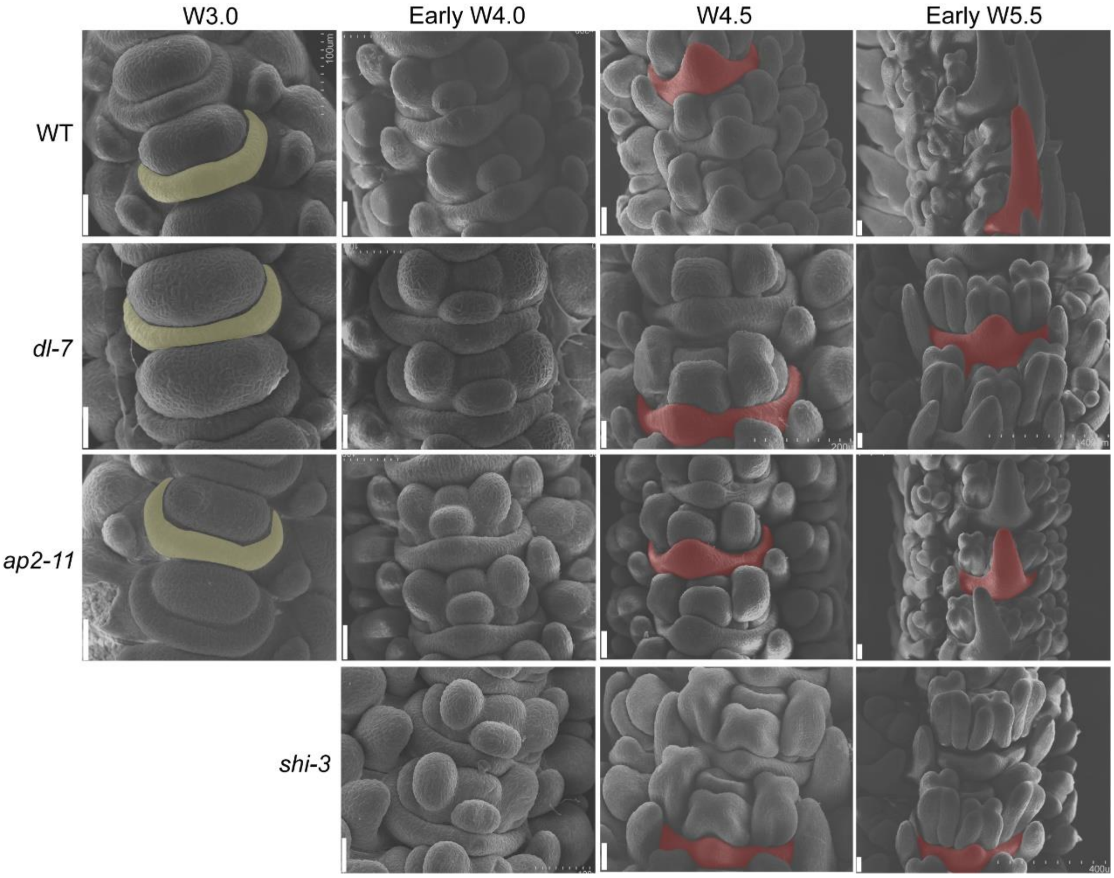


**Supplementary Fig. 8. SEM images of WT, *dl*, *ap2*, and *shi* inflorescence at different developmental stages.**

The yellow and red shading indicate lemma and awn & lemma, respectively. Scale bars, 50 µm. Representative images from three replicates are shown.

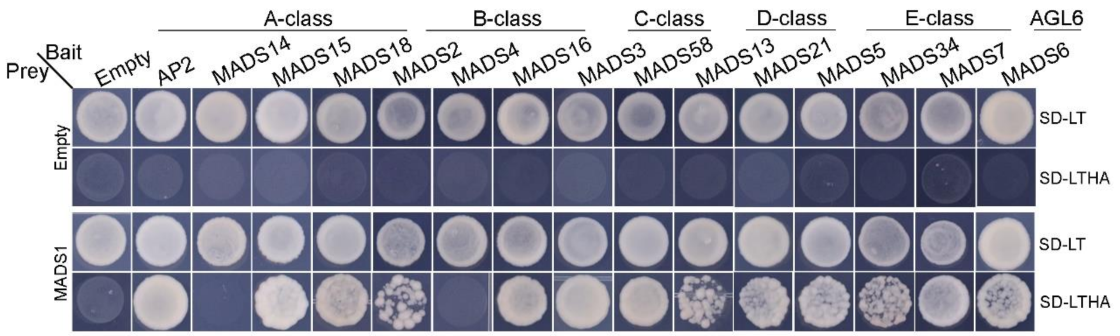


**Supplementary Fig. 9 Interaction of HvMADS1 with other homeotic proteins.**

Y2H assay showing the interaction of HvMADS1 with class A (HvAP2, HvMADS14, HvMADS15), class B (HvMADS2, HvMADS4, HvMADS16), class C (HvMADS3, HvMADS58), class D (HvMADS13, HvMADS21), class E (HvMADS5, HvMADS34, HvMADS7), and AGL6-like (HvMADS6) proteins. A representative result from three independent replicates is shown. Synthetic defined (SD) medium without Trp and Leu (SD/-TL) or without Trp, Leu, His and Ade (SD/-TLHA).

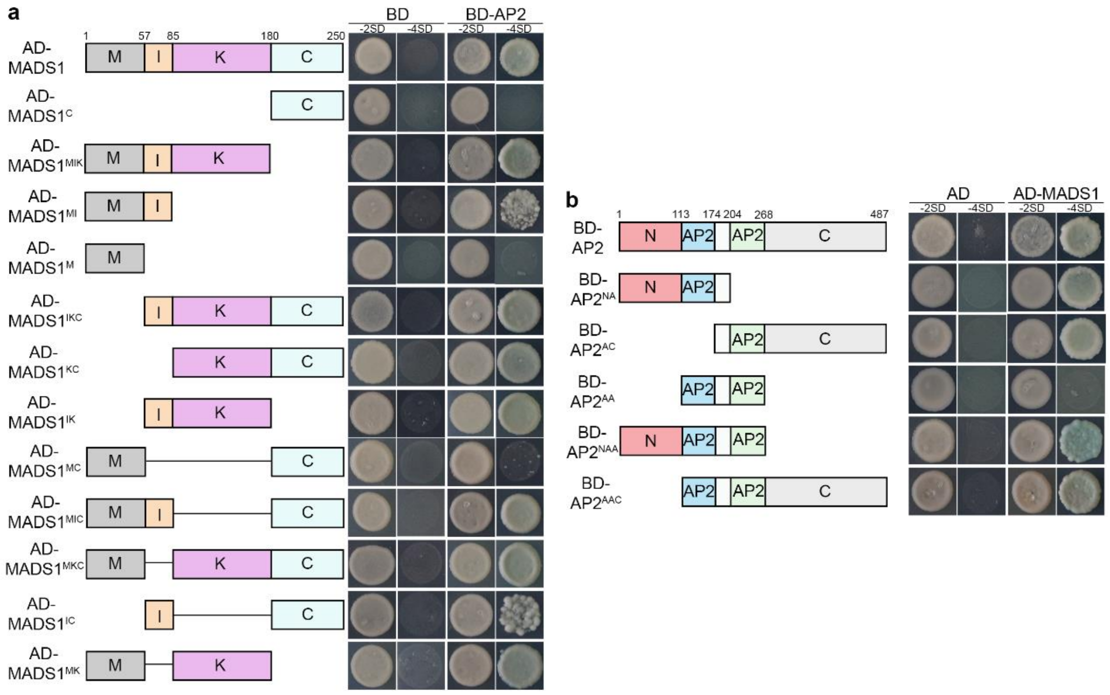


**Supplementary Fig. 10. Dissection of the essential domain for interaction between HvMADS1 and HvAP2 in yeast two-hybrid assays.**

**a** Interaction between different truncated versions of HvMADS1 and intact HvAP2. **b** Interaction between intact HvMADS1 and different truncated versions of HvAP2. A representative result from three independent replicates is shown. AP2, APETALA2 domain; C, C-terminal domain; I, Intervening domain; K, Keratin-like domain; M, MADS domain; N, N-terminal domain. -2SD, SD/-Trp-Leu; -4SD, SD/-Trp-Leu-His-Ade.

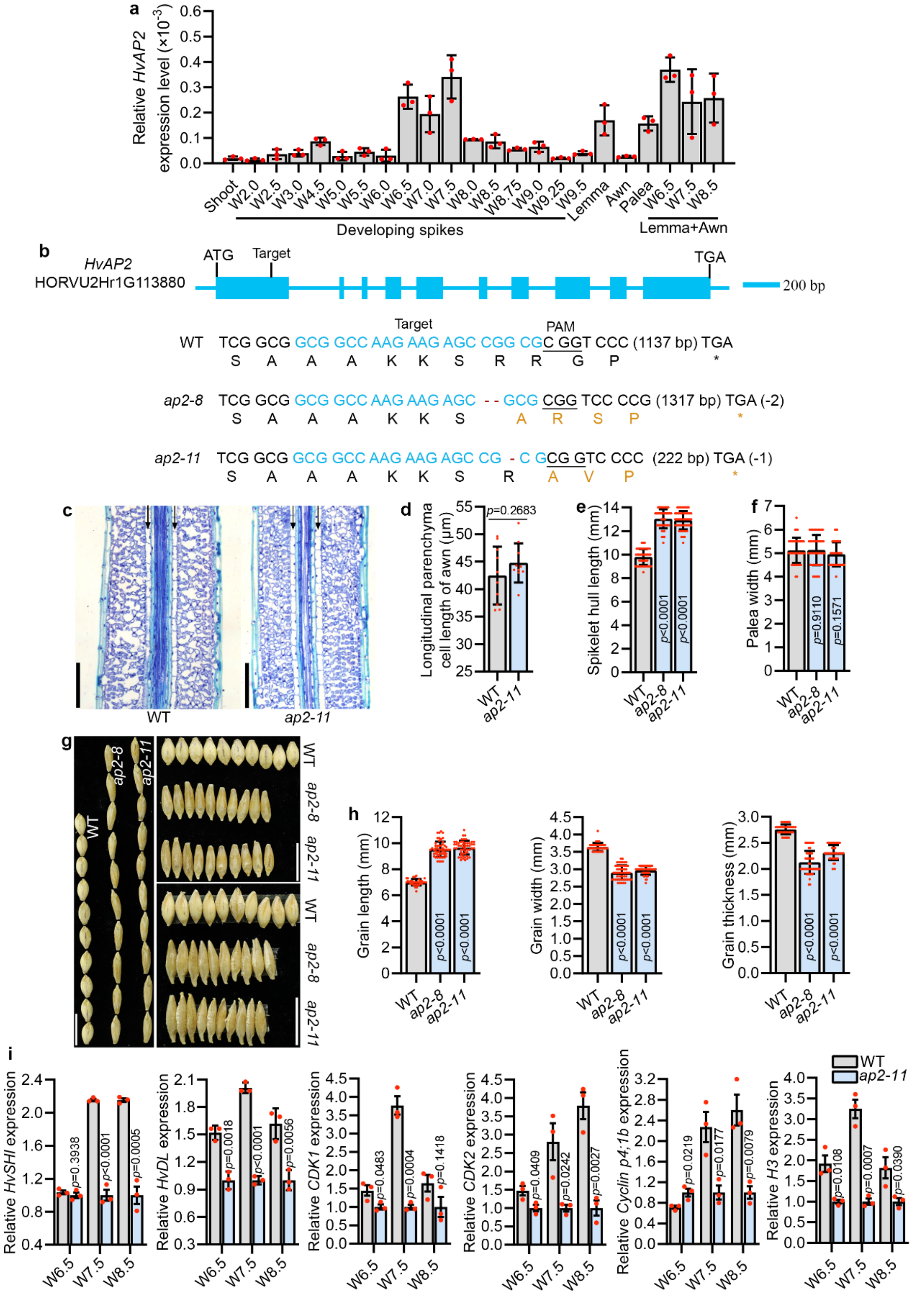

**Supplementary Fig. 11 Phenotypic analysis of *ap2* mutant.**

**a** Spatiotemporal expression patterns of *HvAP2* as revealed by RT-qPCR. **b** Creation of *ap2*

knockout mutant lines using CRISPR/Cas9. Two different mutant alleles were created, *ap2-8* (2- bp deletion, light red dashed line) and *ap2-11* (1- bp deletion, light red dashed line), producing different stop codons (light orange asterisks). **c** Longitudinal section from boxed regions of Fig. 6g, showing cell size of WT (left) and *ap2* (right) awns. Black arrows indicate cells used to measure cell length and cell number. **d–f** Statistic data of longitudinal parenchyma cell length in awns (**d**), spikelet hull length (**e**), and palea width (**f**) of WT and *ap2* plants. **g** Images of WT and *ap2* grains, showing length (left), width (upper right), and thickness (bottom right). **h** Statistic data of length, width, and thickness of WT and *ap2* grains. **i** Expression of *HvSHI*, *HvDL* and four tested cell cycle genes in WT and *ap2* lemmas and awns determined by RT-qPCR. Values are means ± SD, *p* values obtained from two-tailed Student's t-test. Scale bars, 100 μm (**c**), 1 cm (**g**). Source data are provided as a Source Data file.

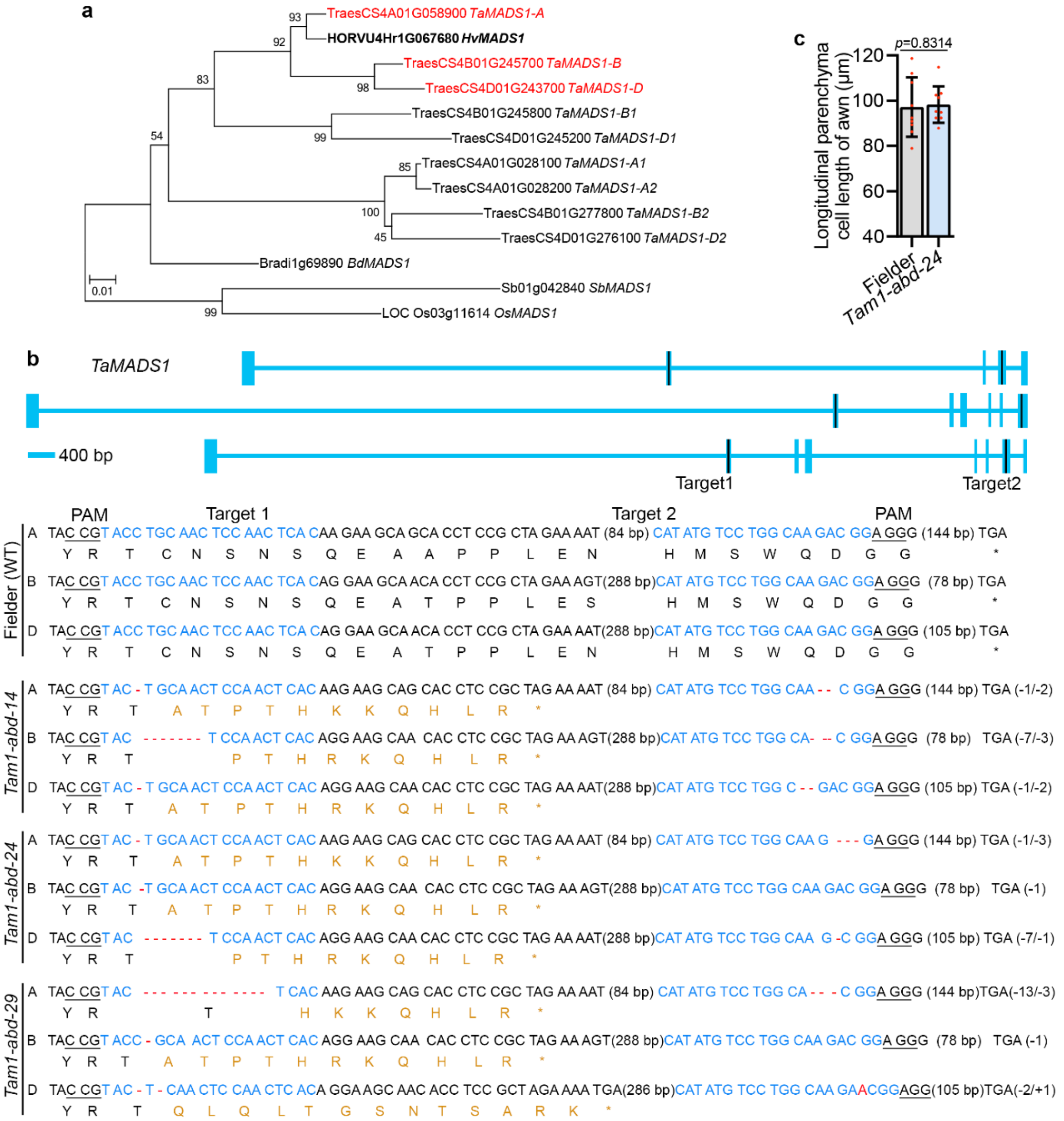


## Supplementary Fig. 12 Characters of *TaMADS1*.

**a** Phylogenetic analysis of *TaMADS1* by the Neighbor-Joining method. *Bd*, *Brachypodium distachyon*; *Hv, Hordeum vulgare; Os, Oryza sativa; Sb, Sorghum bicolor; Ta, Triticum aestivum.* **b** CRISPR/Cas9-mediated mutations in the *TaMADS1-A*, *TaMADS1-B* and *TaMADS1-D* gene loci. Schematic map shows the target sites (Target1 and Target2, light blue letters); PAM, protospacer adjacent motif. Sequences show WT and mutant DNA and predicted amino acid sequences and positions of stop codons (asterisks). **c** Statistic data of the longitudinal parenchyma cell length of WT and *Tam1-abd* awns (n = 9 individual awn samples). Values are means ± SD, *p* values obtained from two-tailed Student's t-test; Source data are provided as a Source Data file.
