# Supplementary material for: MADS1-regulated lemma and awn development benefits barley yield
Source: Nat Commun. 2024 Jan 5;15:301. doi: 10.1038/s41467-023-44457-8 (PMC10770128; doi:10.1038/s41467-023-44457-8)
Supplement: Supplementary file 3 — Description of Additional Supplementary Files [file 41467_2023_44457_MOESM3_ESM.pdf]

Description of Additional Supplementary Files

File Name: Supplementary Data 1

Description: Potential target genes of HvMADS1 revealed by CUT&Tag.

File Name: Supplementary Data 2

Description: The list of 605 genes that were both directly bound by HvMADS1 and differentially expressed in *mads1* mutants identified from CUT&Tag and RNA-seq data.

File Name: Supplementary Data 3

Description: The list of genes related to flower development, phytohormone pathways and cell division for heatmap analysis was revealed from RNA-seq with CUT&Tag data.

File Name: Supplementary Data 4

Description: List of primers used in this study.
